# The Biochemical–Imaging Connection: Urinary Noradrenaline and Fluorodeoxyglucose-Positron Emission Tomography in Unresectable or Metastatic Pheochromocytomas and Paragangliomas

**DOI:** 10.3390/diagnostics15111305

**Published:** 2025-05-22

**Authors:** Junki Takenaka, Shiro Watanabe, Takashige Abe, Satoshi Takeuchi, Kenji Hirata, Rina Kimura, Hiroshi Ishii, Naoto Wakabayashi, Mungunkhuyag Majigsuren, Kohsuke Kudo

**Affiliations:** 1 Department of Diagnostic Imaging, Graduate School of Medicine, Hokkaido University, Sapporo 060-8638, Japan; jtakenaka@pop.med.hokudai.ac.jp (J.T.); khirata@med.hokudai.ac.jp (K.H.); rinakimura343@gmail.com (R.K.); jtjnt011@gmail.com (H.I.); nwakabayashi@pop.med.hokudai.ac.jp (N.W.); ms.mungunkhuyag@gmail.com (M.M.); kkudo@med.hokudai.ac.jp (K.K.); 2 Department of Nuclear Medicine, Hokkaido University Hospital, Sapporo 060-8648, Japan; 3 Global Center for Biomedical Science and Engineering, Faculty of Medicine, Hokkaido University, Sapporo 060-8638, Japan; 4 Department of Renal and Genitourinary Surgery, Graduate School of Medicine, Hokkaido University, Sapporo 060-8638, Japan; takataka@rf6.so-net.ne.jp; 5 Department of Medical Oncology, Faculty of Medicine and Graduate School of Medicine, Hokkaido University, Sapporo 060-8638, Japan; stakeuch@med.hokudai.ac.jp; 6 Center of Neuroendocrine Neoplasms, Hokkaido University Hospital, Sapporo 060-8648, Japan; 7 Medical AI Research and Development Center, Hokkaido University Hospital, Sapporo 060-8648, Japan; 8 Department of Diagnostic and Interventional Radiology, Hokkaido University Hospital, Sapporo 060-8648, Japan; 9 Department of Diagnostic Radiology, National Hospital Organization, Hokkaido Cancer Center, Sapporo 003-0804, Japan; 10 Department of Radiology, Diagnostic Imaging Center, Second State Central Hospital, Ulaanbaatar 210349, Mongolia

**Keywords:** pheochromocytomas, paragangliomas, FDG-PET, catecholamine

## Abstract

**Background/Objectives**: Pheochromocytomas and paragangliomas (PPGLs) are rare tumors of neural crest origin that secrete varying levels of catecholamines. [^18^F]Fluorodeoxyglucose-positron emission tomography (FDG-PET) is a valuable tool for the detection of metastases and the prediction of prognoses. However, varying FDG avidities in PPGLs raise concerns regarding cost-effectiveness and unnecessary radiation exposure. Catecholamine secretion patterns are associated with metastasis and clinical outcomes. This study aimed to explore the relationships among FDG avidity, catecholamine levels, and clinical factors in patients with PPGLs. **Methods**: This retrospective study included 25 patients with unresectable or metastatic PPGLs scheduled for [^131^I]metaiodobenzylguanidine therapy with FDG-PET data available within 40 days of urine catecholamine measurements. FDG avidity was assessed using semiquantitative parameters such as the maximum standardized uptake value (SUVmax), total metabolic tumor volume (MTV), and total lesion glycolysis (TLG). Urine catecholamine levels were quantified. Logistic regression and Spearman’s correlation were performed to evaluate the relationship between FDG parameters and urinary catecholamine levels. **Results**: Urinary noradrenaline levels were significantly higher in patients with FDG-avid lesions than in those without (726.25 μg/day vs. 166.3 μg/day, *p* = 0.001). Noradrenaline levels showed significant positive correlations with SUVmax, MTV, and TLG (ρ = 0.527, 0.541, and 0.557, respectively; all *p* < 0.01). Urinary noradrenaline levels predicted FDG avidity with an AUC of 0.849; a cutoff value of 647.5 μg/day achieved 55.6% sensitivity and 100% specificity. **Conclusions**: Urinary noradrenaline levels were significantly associated with FDG avidity in PPGLs, suggesting their potential utility in predicting FDG-PET outcomes. Therefore, FDG-PET may be unnecessary in PPGL patients with low urinary noradrenaline levels. These findings may help optimize imaging strategies for patients with PPGLs.

## 1. Introduction

Pheochromocytomas and paragangliomas (PPGLs) are rare tumors of neural crest origin that secrete varying levels of catecholamines. According to the 2022 World Health Organization classification, pheochromocytomas are defined as tumors originating in the adrenal medulla, whereas paragangliomas arise from extra-adrenal chromaffin tissue [1]. All PPGLs have the potential to be malignant, and the 5-year survival rate and occurrence of metastases in PPGLs are approximately 75.4–91.0% and 10–30%, respectively [2,3].

Clinical aggressiveness, catecholamine production, genetic mutations, appropriate molecular imaging, and clinical management are interdependently closely related to PPGLs. Recently, PPGLs have been categorized into three clusters based on genetic mutations. Cluster 1 is characterized by gene mutations that stabilize hypoxia-inducible factor-1 (HIF-1), cluster 2 by mutations in tyrosine kinase pathways, and cluster 3 by mutations in the Wnt signaling pathway [4]. Cluster 1 is further subdivided into two subgroups. Cluster 1A includes gene mutations that affect the tricarboxylic acid (TCA) cycle—also known as the Krebs cycle—for example, mutations in succinate dehydrogenase (SDH), isocitrate dehydrogenase (IDH), and fumarate hydratase (FH). These mutations lead to the accumulation of oncometabolites, resulting in pseudohypoxia through the stabilization of HIF-1. In contrast, cluster 1B comprises mutations that directly stabilize HIF-1, such as those in prolyl hydroxylase domain-containing protein (PHD) and the von Hippel–Lindau (VHL) gene [4].

Cluster 3 represents 5–10% of all PPGLs, and is less common than the other clusters. The clinical characteristics of cluster 3 remain poorly understood; therefore, discussions often focus on clusters 1 and 2. Cluster 1A tumors exhibit the highest metastatic potential, and are considered the most aggressive, highlighting the need for prompt intervention [5,6,7,8]. These tumors typically exhibit noradrenergic or dopaminergic phenotypes [9,10]. Cluster 1B tumors have an intermediate metastatic risk [8,11,12,13], and exhibit a noradrenergic phenotype [14]. Cluster 2 tumors are less metastatic, and primarily exhibit an adrenergic phenotype [14].

Catecholamine biosynthesis involves the enzymatic conversion of tyrosine into dihydroxyphenylalanine (DOPA), followed by conversions into dopamine, noradrenaline, and adrenaline, wherein noradrenergic and dopaminergic phenotypes are indicative of enzymatic defects and are considered less mature than the adrenergic type [14,15].

Some PPGLs demonstrate poor metaiodobenzylguanidine (MIBG) uptake [16] while overexpressing somatostatin receptors (SSTRs), a characteristic commonly associated with tumors classified in cluster 1A [17]. Consequently, [^123^I]MIBG scintigraphy and [^131^I]MIBG therapy are less effective for these tumors [16,17]. In contrast, SSTR positron emission tomography (PET) imaging (e.g., [^68^Ga]/[^64^Cu]DOTATATE PET) is superior, and peptide receptor radionuclide therapy (PRRT) has emerged as a highly effective therapy for PPGLs in this cluster [18,19,20,21]. Severi et al. reported that PRRT is well tolerated by patients, has no significant renal or bone marrow toxicity, and achieves a disease control rate of 80% [22]. Clusters 1B and 2, on the other hand, overexpress norepinephrine or L-type amino acid transporters, but only weakly express SSTRs [23,24]. [^18^F]FDOPA PET/computed tomography (CT) is the first-choice tracer for detecting metastasis, and [^131^I]MIBG is one of the most effective systemic treatments [25,26,27,28,29,30,31,32].

[^18^F]Fluorodeoxyglucose (FDG)-PET is not the first-line recommended tracer for evaluating metastasis, but is generally used to detect metastatic lesions in various tumors [33]. Furthermore, FDG-PET facilitates prognosis [34] and response assessment [35]. Notably, FDG-PET positivity of PPGL metastases, particularly when not corresponding to SSTR expression, may represent an exclusion criterion for PRRT, because such lesions may reflect dedifferentiated, more aggressive phenotypes with reduced therapeutic benefit from PRRT [36]. In a prospective study of various neuroendocrine neoplasms (including PPGLs), FDG uptake was significantly decreased after PRRT, particularly with a dual-radionuclide scheme, indicating that FDG-PET may also serve as a dynamic biomarker for treatment responses [37]. However, as the FDG avidity of PPGLs varies, the effectiveness of FDG-PET also varies. In a previous report, PPGLs in cluster 1A and 1B showed high FDG avidity, whereas those in cluster 2 showed low avidity [33]. This may be due to enhanced glycolysis resulting from defects in the TCA cycle or activation of HIF-1 in cluster 1 caused by genetic mutations that are not observed in cluster 2 [4,36,38]. By analyzing genetic mutations, it may be possible to predict FDG uptake. However, examining mutations in these tumors is ethically and practically challenging, although these results may have significant implications not only for patients, but also for their biological relatives. Therefore, this study aimed to explore the predictive factors for FDG avidity in patients with PPGLs.

## 2. Materials and Methods

### 2.1. Patients

This retrospective study was approved by the Institutional Review Board of Hokkaido University Hospital (approval number: 022-0329, approved on 13 February 2025) and conducted in accordance with the principles outlined in the World Medical Association’s Declaration of Helsinki. The requirement for written informed consent was waived due to the retrospective nature of the study.

**Figure 1:**
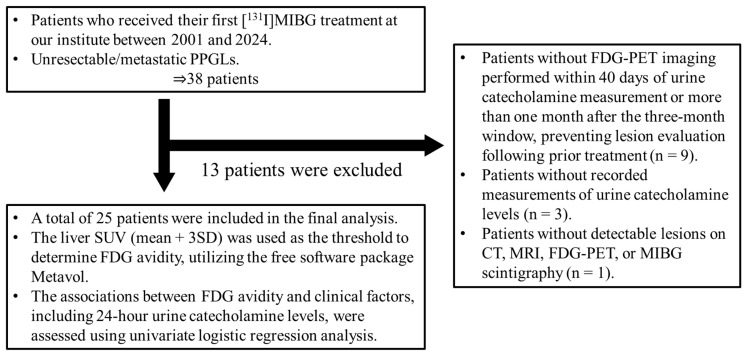
Flow diagram of the participant inclusion process. CT, computed tomography; FDG, [^18^F]fluorodeoxyglucose; MIBG, metaiodobenzylguanidine; MRI, magnetic resonance imaging; PET, positron emission tomography; PPGLs, pheochromocytomas and paragangliomas; SD, standard deviation; SUV, standardized uptake value.

We retrospectively reviewed 38 patients with unresectable or metastatic PPGLs who received [^131^I]MIBG therapy at our institution between 2001 and 2024. Of these, 9 patients were excluded due to the absence of FDG-PET data within 40 days of urinary catecholamine measurement, 3 patients were excluded due to missing urinary catecholamine data, and 1 patient was excluded because no detectable lesions were found on CT, MRI, FDG-PET, or MIBG scintigraphy. As a result, 25 patients were included in the final analysis (Figure 1).

### 2.2. FDG-PET Acquisition

All scans were performed at our institution, spanning 20 years during which four PET or PET/CT scanners were used for FDG-PET examinations in this patient cohort. From 2001 to 2006, two PET scanners (ECAT EXACT 47 and ECAT EXACT HR+; Siemens Healthineers, Erlangen, Germany) were used for FDG-PET prior to [^131^I]MIBG therapy. After 2006, two PET/CT scanners (Biograph 64 TruePoint, Siemens Healthineers; GEMINI TF64, Philips Healthcare, Amsterdam, The Netherlands) were used. FDG-PET and PET/CT imaging were performed according to standard clinical practice.

Patients were instructed to fast for at least 6 h prior to FDG-PET/CT imaging. PET scans were acquired 60 min after intravenous administration of FDG at a dose of 4 MBq/kg. For the stand-alone PET scanners, imaging included a 2 min-per-bed emission scan and a 2 min-per-bed transmission scan using a Ge-68/Ga-68 source for attenuation correction. Images were reconstructed using the ordered subset expectation maximization (OSEM) algorithm with one iteration and 30 subsets. For PET/CT systems, emission scans were acquired following CT-based attenuation correction. Reconstruction settings were as follows: Biograph 64 TruePoint (3D-OSEM) with two iterations and 21 subsets, and GEMINI TF64 (3D-OSEM) with three iterations and 33 subsets.

### 2.3. Image Analysis

We used the open-source software Metavol [39] to evaluate FDG avidity, specifically to assess whether lesion uptake exceeded liver uptake. A semi-automated 3 cm-diameter volume of interest (VOI) was placed in the right hepatic lobe to calculate the mean and standard deviation (SD) of FDG uptake. The criteria for FDG avidity were established using a threshold of the mean + 3 SD to ensure consistency [40]. Patients with at least one FDG-avid lesion were classified into the FDG-avid group, whereas those with no FDG-avid lesion were assigned to the non-avid group.

In this study, we used three semiquantitative parameters for FDG-PET: maximum standardized uptake value (SUVmax), total metabolic tumor volume (MTV), and total tumor lesion glycolysis (TLG). The standardized uptake value (SUV) was calculated using the following equation:

SUV = c · w/d,

where c represents the FDG concentration in a specific voxel (Bq/mL), w denotes the patient’s body weight (g), and d is the decay-corrected dose of injected FDG (Bq). Assuming a body density of 1 g/mL, the SUV was treated as a dimensionless value. MTV was defined as the total volume within the tumor boundaries, whereas TLG was calculated as the product of the mean SUV within the tumor and MTV.

The threshold of mean + 3 SD was consistently applied to determine tumor boundaries, with voxels exceeding this threshold across the entire body being automatically extracted and highlighted. A nuclear medicine physician (J.T., with 9 years of experience in PET interpretation) initially reviewed the highlighted regions to exclude physiological uptake in non-tumor tissues (e.g., brain, myocardium, urinary tract), referring to corresponding CT and MRI findings. These assessments were subsequently confirmed by a second nuclear medicine physician (S.W., with 14 years of experience), and any discrepancies were resolved through consensus. In cases where the tumor and non-tumor regions were contiguous, the non-tumor areas were carefully excluded using Metavol’s manual polygon-shaped region-of-interest tool. Semiquantitative parameters (SUVmax, MTV, and TLG) were defined as 0 in patients belonging to the non-avid group. The highest SUVmax, as well as the total MTV and TLG, were recorded for each patient.

As two of the four PET scanners employed in this study were decommissioned and unavailable for further analyses, images from all four scanners acquired using different reconstruction methods were collectively analyzed without standardization or harmonization.

### 2.4. Biochemical Parameters

Urinary levels of adrenaline, noradrenaline, and dopamine were measured from 24 h urine collections preserved in hydrochloric acid. Excess catecholamine secretion was defined as any urinary catecholamine level exceeding the upper limit of the normal range.

### 2.5. Statistical Analyses

Statistical analyses were performed using JMP^®^ version 17.0 (SAS Institute, Cary, NC, USA). Univariate logistic regression was used to assess clinical variables—such as age at initial treatment, sex, disease type (PPGL), prior chemotherapy and external radiotherapy, number of organs with metastatic lesions, and 24 h urinary catecholamine levels—as potential predictors of FDG avidity in metastatic lesions. The diagnostic performance of significant predictive factors was further assessed using receiver operating characteristic (ROC) curve analysis. Correlations between urinary catecholamine levels and FDG-PET semiquantitative parameters were examined using Spearman’s rank correlation coefficients. Statistical significance was set at *p* < 0.05.

## 3. Results

### 3.1. Patient Characteristics

The patient characteristics are summarized in Table 1. Among the enrolled 25 patients, 17 (68.0%) had pheochromocytomas, and the remaining 8 (32.0%) had paragangliomas. In total, 11patients (44.0%) were male, including 6 of 17 (35.3%) patients with pheochromocytoma and 5 of 8 (62.5%) patients with paraganglioma. The median age at the time of initial [^131^I]MIBG treatment at our institute was 53.0 (range: 23–84) years, with a median of 54.0 (range: 35–84) years for patients with pheochromocytoma and 48.5 (range: 23–67) years for patients with paraganglioma. In total, 18 (72.0%) patients had FDG-avid lesions.

Pre-treatment history included surgery in 24 patients (96.0%), chemotherapy in 8 (32.0%), and external radiation in 7 (28.0%). Among them, 15 of 17 (88.2%) patients with pheochromocytoma, and all (100.0%) patients with paraganglioma underwent surgery. Chemotherapy was given to six (35.3%) patients with pheochromocytoma and two (25.0%) patients with paraganglioma; external radiation was administered to five (29.4%) and two (25.0%) patients, respectively. One (12.5%) patient with paraganglioma had received prior [^131^I]MIBG therapy at another hospital (dose: 7.4 GBq). Additionally, two (8.0%) patients underwent radiofrequency ablation (pheochromocytoma and paraganglioma in one patient each), and one patient (4.0%) received endovascular treatment.

Imaging showed tumor evidence in all patients, including residual primary tumors in two patients (pheochromocytoma and paraganglioma in one patient each). Metastatic sites included lymph nodes or soft tissue (14 patients, 56.0%), bones (14, 56.0%), liver (12, 48.0%), and lungs (10, 40.0%). Patients with pheochromocytoma more frequently had liver (58.8%) and bone (64.7%) metastases than those with paraganglioma (25.0% and 37.5%, respectively). The number of metastatic organs was one in nine patients (36.0%), two in ten patients (40.0%), three in four patients (16.0%), and four in two patients (8.0%). The distribution across tumor types showed that 50.0% of patients with paraganglioma had metastases in only one organ, whereas 11.8% of patients with pheochromocytoma had metastases in four organs.

Regarding excess catecholamine secretion, increased urinary levels of adrenaline, noradrenaline, and dopamine were observed in 3 (12.0%), 20 (80.0%), and 13 (52.0%) patients, respectively. Among them, adrenaline excess was only seen in patients with pheochromocytoma (17.6%), whereas excessive noradrenaline and dopamine levels were more common in patients with paragangliomas (87.5% and 62.5%, respectively).

**Figure 2:**
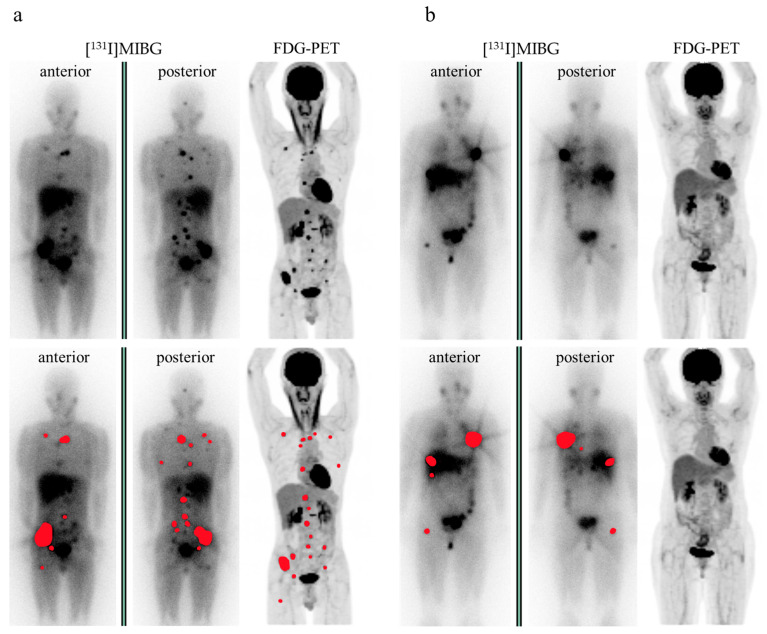
Representative cases. (**a**) A patient with bone and lymph node metastases. The treatment-dose [^131^I]MIBG scintigraphy (planner image) and the FDG-PET (maximum intensity projection [MIP] image) are shown. Metastatic lesions are indicated in red. Urinary catecholamine levels were as follows: adrenaline, 15.6 μg/day; noradrenaline, 3991 μg/day; and dopamine, 906.1 μg/day. All metastatic lesions demonstrated marked FDG avidity. (**b**) A patient with bone, liver, and lung metastases. The planner image of [^131^I]MIBG scintigraphy and the MIP image of FDG-PET are shown. Metastatic lesions are highlighted in red. Urinary catecholamine levels were as follows: adrenaline, 18.3 μg/day; noradrenaline, 179 μg/day; and dopamine, 963.2 μg/day. None of the metastatic lesions showed significant FDG uptake. MIP, maximum intensity projection.

Two representative cases are illustrated in Figure 2.

### 3.2. Biochemical Parameters

The 24 h urine samples were collected at a median of 2 days after FDG-PET (range: −34 to 38 days). As summarized in Table 2, the median urinary adrenaline level was 6.9 μg/day (range: 1.8–1080 μg/day; interquartile range [IQR]: 3.35–13.8 μg/day; normal range: 3.4–26.9 μg/day).

When stratified by FDG avidity, the median adrenaline levels were 7.7 μg/day (range: 2.3–1080 μg/day; IQR: 5.0–14.6 μg/day) in the FDG-avid group and 3.4 μg/day (range: 1.8–18.3 μg/day; IQR: 3.0–11.6 μg/day) in the non-avid group.

The median urinary noradrenaline level was 224.3 μg/day (range: 59.1–4282 μg/day; IQR: 172.9–945.95 μg/day; normal range: 48.6–168.4 μg/day). In subgroup analyses, the FDG-avid group showed a higher median noradrenaline level of 726.25 μg/day (range: 155–4020.1 μg/day; IQR: 186.7–1275.9 μg/day), compared to 166.3 μg/day (range: 59.1–273.9 μg/day; IQR: 86.1–224.3 μg/day) in the non-avid group.

The median urinary dopamine level was 963.2 μg/day (range: 249.9–3187.5 μg/day; IQR: 644.95–1459.35 μg/day; normal range: 365.0–961.5 μg/day). The FDG-avid group had a median dopamine level of 1050.3 μg/day (range: 379.7–3187.5 μg/day; IQR: 658.2–1637.7 μg/day), whereas the non-avid group had a lower median of 819.4 μg/day (range: 249.9–1426.3 μg/day; IQR: 495.3–1190.4 μg/day).

**Figure 3:**
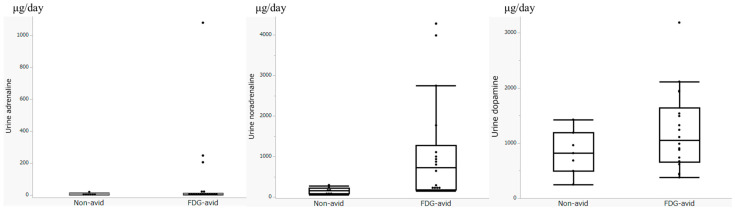
Box plots of the urinary catecholamine levels in the FDG-avid and non-avid groups.

Figure 3 shows box plots illustrating all urinary catecholamine levels in the FDG-avid and non-avid groups.

### 3.3. FDG-PET Imaging Parameters

The semiquantitative analysis in the 18 patients with FDG-avid lesions resulted in the following median values: SUVmax, 10.65 (range: 4.64–64.45; IQR: 7.425–42.51); MTV, 83.54 (range: 1.21–395.31; IQR: 19.32–118.82); and TLG, 396.65 (range: 4.50–4017.35; IQR: 80.10–752.49; Table 3).

### 3.4. Evaluation of FDG Avidity and Associated Clinical Data, Including Urinary Catecholamine Levels

The median urinary noradrenaline level was significantly higher in patients with FDG-avid lesions (726.25 μg/day; range: 155–4020.1 μg/day) than in those with non-avid lesions (166.3 μg/day; range: 59.1–273.9 μg/day). Excess noradrenaline secretion was observed in 17 of 18 patients with FDG-avid lesions, compared to only three of seven patients with non-avid lesions. Excess noradrenaline secretion was significantly associated with FDG avidity (odds ratio: 22.66, 95% confidence interval: 2.399–541.7, *p* = 0.005). In addition, patients with FDG-avid lesions were more likely to have a history of external beam radiation therapy than those without FDG-avid lesions. Higher urinary noradrenaline levels (odds ratio: 1.013, *p* = 0.001) and a history of external radiation (odds ratio: uncalculatable, *p* = 0.018) were identified as significant predictive factors for FDG avidity (Table 4), whereas other clinical factors were not associated with FDG avidity.

**Figure 4:**
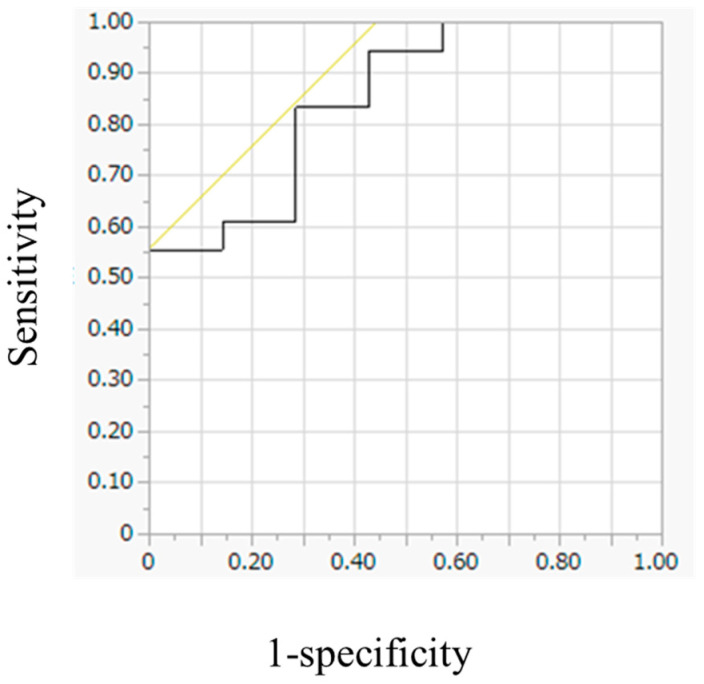
Receiver operating characteristic curves for urinary noradrenaline levels. The cutoff value is 647.5 μg/day (area under the curve: 0.849), with a sensitivity of 55.6% and a specificity of 100.0%.

The optimal cutoff value for urinary noradrenaline to predict FDG avidity was determined using ROC curve analysis (Figure 4). Urinary noradrenaline levels predicted FDG avidity with an area under the curve (AUC) of 0.849; a cutoff value of 647.5 μg/day achieved 55.6% sensitivity and 100% specificity.

**Figure 5:**
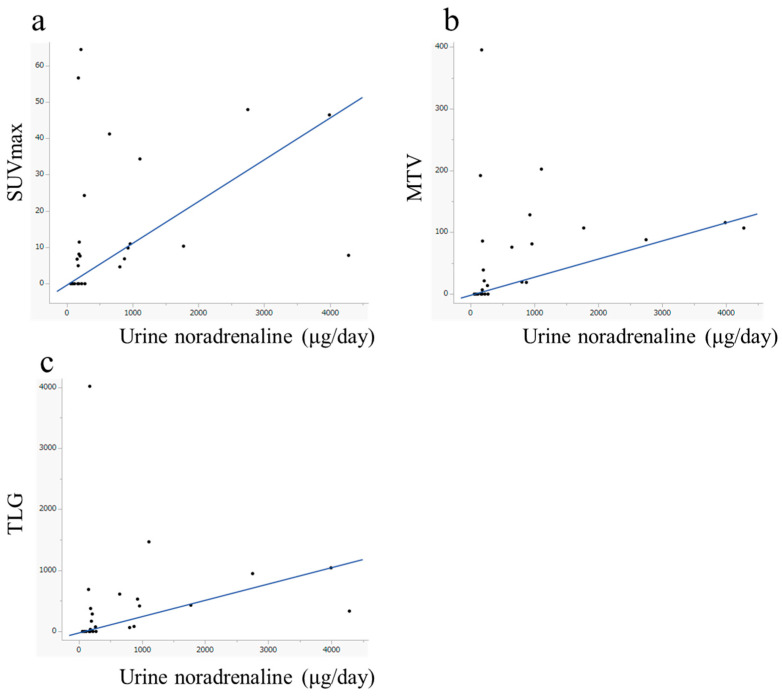
Scatter plots with regression lines illustrating the relationships between urinary noradrenaline levels and (**a**) SUVmax, (**b**) MTV, and (**c**) TLG. Urinary noradrenaline levels showed significant correlations with SUVmax (ρ = 0.527, *p* = 0.007), MTV (ρ = 0.541, *p* = 0.004), and TLG (ρ = 0.557, *p* = 0.004).

Urinary noradrenaline levels showed significant correlations with SUVmax (ρ = 0.527, *p* = 0.007), MTV (ρ = 0.541, *p* = 0.004), and TLG (ρ = 0.557, *p* = 0.004). No statistically significant correlations were observed between other catecholamines and any combination of semiquantitative FDG-PET parameters (Figure 5, Table 5).

## 4. Discussion

In this retrospective analysis of 25 patients with PPGLs, we investigated the clinical factors predicting FDG avidity. Our findings provide insights that may help to avoid unnecessary FDG-PET imaging of patients with PPGLs.

PPGLs are categorized into three main molecular clusters: pseudohypoxia cluster 1 (subdivided into 1A and 1B), kinase signaling cluster 2, and Wnt signaling cluster 3. The Wnt signaling cluster 3 is relatively rare; thus, its clinical, pathological, and genetic characteristics are often discussed in comparison with those of the pseudohypoxia cluster 1 (1A and 1B) and the kinase signaling cluster 2 [4]. It has been suggested that patients with cluster 1 PPGLs not only exhibit a higher frequency of metastasis [8], but also have a poorer prognosis [41]. Pseudohypoxia cluster 1 is further divided into clusters 1A and 1B, based on the location of the genetic mutation. Mutations related to the Krebs cycle, such as SDH and IDH mutations, are classified as cluster 1A, whereas mutations in hypoxia-signaling pathways, such as VHL mutations, are classified as cluster 1B. In cluster 1A, defects in the Krebs cycle lead to an increased expression of glycolytic enzymes [36]. In cluster 1B, stabilization of HIF-1α by the hypoxia-signaling pathway activates glycolysis and suppresses the Krebs cycle [4,38]. Consequently, the PPGLs in clusters 1A and 1B are more FDG-avid than those in cluster 2 [4,33]. Clinically, most cluster 1 PPGLs exhibit a noradrenergic phenotype, and cluster 1A PPGLs produce not only noradrenaline but also dopamine [9], which aligns with our finding that FDG avidity is significantly associated with urinary noradrenaline levels. However, a previous study reported a significant relationship between metanephrine (a metabolite of adrenaline) levels and SUV [33]. Furthermore, some studies have indicated that FDG avidity is not associated with patterns of catecholamine production [42]. These findings are inconsistent with our results, and may be attributed to the inclusion of a large number of primary tumor cases. This discrepancy might be explained by differences in catecholamine production patterns between primary tumors and metastases, even within the same patient [43].

Moreover, we demonstrated that MTV and TLG, which are indicators of tumor volume, were significantly associated with urinary noradrenaline levels. We have previously reported that the group with higher MTV/TLG has a poorer prognosis [34]. MTV/TLG are considered to reflect not only simple tumor volume, but also the pseudohypoxia cluster. However, the correlation between noradrenaline and MTV/TLG was not strong, suggesting that FDG-PET and catecholamine measurements were complementary. Therefore, our findings indicate that FDG-PET and catecholamine levels should be evaluated separately.

In our study, noradrenaline emerged as a useful indicator for predicting FDG avidity, with the optimal cutoff value achieving 55.6% sensitivity and 100% specificity. Patients with a history of PPGL require lifelong follow-up [4]. In addition to imaging, periodic biochemical monitoring—particularly catecholamine assessment—is essential. Our findings suggest that in the absence of elevated noradrenaline levels, FDG-PET may be unnecessary, potentially avoiding superfluous radiation exposure and reducing the number of unwarranted imaging examinations.

However, the false-negative rate was relatively high. Patients in the false-negative group tended to exhibit higher dopamine levels (median: 1409 ng/mL, range: 444.6–3187.5) compared to patients with FDG-avid tumors (median: 880.9 ng/mL, range: 249.9–2115.9). Although dopamine levels may also influence FDG avidity, this study did not provide definitive evidence to support this association because additional analyses could not be performed, owing to the limited sample size and the issue of multiple comparisons.

FDG-avid lesions can be evaluated following PRRT and [^131^I]MIBG therapy [35,37]. Notably, bone metastases often cannot be assessed using RECIST criteria [44]. Moreover, changes in FDG uptake in response to [^131^I]MIBG treatment may become apparent earlier than changes in tumor size observed on CT [45]. Therefore, FDG avidity plays a crucial role in the early assessment of treatment response.

In this study, a higher proportion of patients in the FDG-avid group had a history of external beam radiation therapy than those in the non-avid group. This finding is likely associated with an increased risk of skeletal-related events (SREs). Previous studies have indicated that a history of [^131^I]MIBG radionuclide therapy and the absence of liver metastases are factors that reduce the SRE risk [46]. SREs are estimated to be linked to classification within poor prognosis groups such as cluster 1; however, specific predictive factors for SRE have not yet been clearly identified [47]. A history of radiation therapy is considered to be associated with more aggressive tumor behavior, which may correlate with the presence of FDG-avid lesions.

FDG-PET imaging is generally useful for detecting metastases in patients with PPGLs that are not clearly identified by conventional imaging modalities (such as CT or MRI) or by other functional imaging techniques (such as MIBG or SSTR-PET). Based on our observations, patients with normal urinary noradrenaline levels may derive less benefit from FDG-PET imaging compared to those with elevated levels. However, it remains unclear whether gene mutations associated with FDG avidity directly stimulate noradrenaline production. Catecholamines are synthesized via a cascade of enzymatic reactions, beginning with the conversion of tyrosine to DOPA, followed by sequential transformation into dopamine, noradrenaline, and adrenaline [48]. Based on their biochemical profiles, PPGLs can be divided into adrenergic, noradrenergic, and dopaminergic phenotypes. The noradrenergic and dopaminergic phenotypes likely correspond to the pseudohypoxia cluster 1, which is associated with enzyme defects and is considered less mature than the adrenergic phenotype [15,48,49]. However, these gene mutations are not known to directly cause enzyme defects.

[^68^Ga]/[^64^Cu]DOTATATE PET is currently the first-choice imaging modality for detecting metastases because of its high sensitivity and specificity [4,21,36]. FDG-PET, which has a comparatively lower sensitivity and specificity, is recommended only when SSTR-PET is unavailable. Furthermore, SSTR-PET is essential for planning and performing PRRT [4,36,50]. Additional imaging modalities, including [^123^I]MIBG scintigraphy [4,27,29,51], [^124^I]MIBG-PET [52], and [^18^F]MFBG-PET [53] are valuable for detecting metastases and determining their suitability for therapy. Although FDG-PET cannot determine the eligibility for radionuclide therapy and has a lower sensitivity and specificity than SSTR-PET, it remains crucial for PPGLs because of its wide availability. Additionally, FDG-PET may be useful for classifying cluster 1 tumors and differentiating them from tumors of other clusters.

### Study Limitations

This study was a single-center retrospective analysis with a limited sample size (n = 25), collected over a 20-year period. The extended duration was necessary due to the extreme rarity of PPGLs, particularly those that are unresectable or metastatic. The small number of cases limited statistical power and precluded the use of multivariate analysis. Further multicenter studies are required to validate these findings and enhance the reliability of the diagnostic and monitoring strategies for PPGLs.

Only patients scheduled to undergo [^131^I]MIBG therapy were included. In Japan, MIBG therapy has long been the first-line treatment for unresectable and metastatic MIBG-avid PPGLs. Therefore, we believe that most cases were covered, with the exception of those facing barriers to accessing treatment.

Semiquantitative parameters from multiple PET scanners were analyzed without standardization or harmonization, as some of the scanners were no longer available at our institution. To mitigate interscanner variability, liver uptake was used as a reference to determine FDG avidity, thereby minimizing differences in semiquantitative measurements as much as possible.

Catecholamine metabolites, including metanephrine, normetanephrine, and 3-methoxytyramine, were not assessed in this study. These metabolites are continuously produced by PPGLs and exhibit fewer physiological fluctuations than catecholamines. Consequently, they are considered reliable biomarkers for the diagnosis and monitoring of PPGLs [4,54,55]. However, the measurement of 3-methoxytyramine is still clinically unavailable in Japan, and metanephrine and normetanephrine were not analyzed in this study owing to significant missing data as their measurements were not approved until 2019.

Genetic mutations such as SDH and VHL mutations were not evaluated in this study because of the ethical and practical challenges associated with examining germline mutations. These findings may carry important implications for both patients and their blood relatives. Future studies should address these limitations to enhance the validity of our findings.

## 5. Conclusions

Our findings indicate that it may be possible to estimate FDG avidity by using urine noradrenaline levels in patients with PPGLs. FDG-PET might be unnecessary in PPGL patients with low urinary noradrenaline levels. In the future, it may be possible to determine FDG indications in patients with PPGLs without examining germline mutations.

## Figures and Tables

**Table 1:** Patient characteristics.

Characteristic	All	Pheochromocytoma	Paraganglioma
Number	25	17	8
Sex			
Male	11 (44.0%)	6 (35.3%)	5 (62.5%)
Female	14 (56.0%)	11 (64.7%)	3 (37.5%)
Age (y), median (range)	53 (23–84)	54 (35–84)	48.5 (23–67)
Pre-treatment			
Surgery	24 (96.0%)	15 (88.2%)	8 (100.0%)
Chemotherapy	8 (32.0%)	6 (35.3%)	2 (25.0%)
External radiation	7 (28.0%)	5 (29.4%)	2 (25.0%)
MIBG	1 (4.0%)	0 (0.0%)	1 (12.5%)
Radiofrequency ablation	2 (8.0%)	1 (5.9%)	1 (12.5%)
Endovascular treatment	1 (4.0%)	1 (5.9%)	0 (0.0%)
Residual primary tumor	2 (8.0%)	1 (5.9%)	1 (12.5%)
Metastasis			
Lymph node or soft tissue	14 (56.0%)	9 (52.9%)	5 (62.5%)
Bone	14 (56.0%)	11 (64.7%)	3 (37.5%)
Liver	12 (48.0%)	10 (58.8%)	2 (25.0%)
Lung	10 (40.0%)	7 (41.2%)	3 (37.5%)
Metastasis (no. of organs):			
1	9 (36.0%)	5 (29.4%)	4 (50.0%)
2	10 (40.0%)	7 (41.2%)	3 (37.5%)
3	4 (16.0%)	3 (17.6%)	1 (12.5%)
4	2 (8.0%)	2 (11.8%)	0 (0.0%)
Excess catecholamine secretion			
Adrenaline	3 (12.0%)	3 (17.6%)	0 (0.0%)
Noradrenaline	20 (80%)	13 (76.5%)	7 (87.5%)
Dopamine	13 (52.0%)	8 (47.1%)	5 (62.5%)
Patients with FDG-avid lesions	18 (72.0%)	12 (70.6%)	6 (75.0%)

Data are presented as n (%).

**Table 2:** Urinary biochemistry parameters (μg/day).

		Median	Range	IQR	Normal Range
Adrenaline	All	6.9	1.8–1080	3.35–13.8	3.4–26.9
	FDG-avid	7.7	2.3–1080	5.0–14.6	
	Non-avid	3.4	1.8–18.3	3.0–11.6	
Noradrenaline	All	224.3	59.1–4282	172.9–945.95	48.6–168.4
	FDG-avid	726.25	155–4020.1	186.7–1275.9	
	Non-avid	166.3	59.1–273.9	86.1–224.3	
Dopamine	All	963.2	249.9–3187.5	644.95–1459.35	365.0–961.5
	FDG-avid	1050.3	379.7–3187.5	658.2–1637.7	
	Non-avid	819.4	249.9–1426.3	495.3–1190.4	

IQR, interquartile range.

**Table 3:** FDG-PET semiquantitative parameters in patients with FDG-avid lesions.

	Median	Range	IQR
SUVmax	10.65	4.64–64.45	7.425–42.51
MTV	83.54	1.21–395.31	19.32–118.82
TLG	396.65	4.50–4017.35	80.10–752.49

MTV, metabolic tumor volume; SUVmax, standardized uptake value; TLG, total lesion glycolysis.

**Table 4:** Univariate logistic regression analysis comparing patients with and without FDG-avid lesions.

Variable	With FDG-avid Lesions	Without FDG-avid Lesions	OR (95% CI)	*p*-Value
Number	18	7		
Sex				
Male	9	2	1.620 (0.335–8.230)	0.548
Female	9	5		
Age (y), median (range)	61 (23–84)	47 (35–53)	1.047 (0.984–1.128)	0.150
Diagnosis				
Pheochromocytoma	12	5	0.800 (0.095–5.071)	0.818
Paraganglioma	6	2		
History of chemotherapy	4	4	0.214 (0.030–1.341)	0.100
History of external radiation	7	0	>100	0.018 *
Metastatic lesion				
Lymph node or soft tissue	12	2	5.000 (0.815–42.97)	0.083
Bone	11	3	2.095 (0.357–13.58)	0.410
Liver	8	4	0.600 (0.098–3.501)	0.568
Lung	8	2	2.000 (0.329–16.67)	0.461
Urine biochemistry				
Excess adrenaline secretion	3	0	>100 (0.448–NA)	0.145
Adrenaline (μg/day), median (range)	7.7 (2.3–1080)	3.4 (1.8–18.3)	1.068 (0.999–1.347)	0.110
Excess noradrenaline secretion	17	3	22.66 (2.399–541.7)	0.005 *
Noradrenaline (μg/day), median (range)	726.25 (155–4020.1)	166.3 (59.1–273.9)	1.013 (1.002–1.039)	0.001 *
Excess dopamine secretion	10	3	1.667 (0.286–10.66)	0.568
Dopamine (μg/day), median (range)	1050.3 (379.7–3187.5)	819.4 (249.9–1426.3)	1.001 (1.000–1.004)	0.145

CI, confidence interval; NA, not available; OR, odds ratio. * *p* ≤ 0.05.

**Table 5:** FDG-PET semiquantitative parameters.

	Urine Biochemistry	ρ	*p*-Value
SUVmax	Adrenaline	0.246	0.236
	Noradrenaline	0.527	0.007 *
	Dopamine	0.369	0.069
MTV	Adrenaline	0.274	0.185
	Noradrenaline	0.541	0.004 *
	Dopamine	0.299	0.147
TLG	Adrenaline	0.296	0.151
	Noradrenaline	0.557	0.004 *
	Dopamine	0.336	0.101

* *p* ≤ 0.05.

## Data Availability

The data presented in this study are available from the corresponding author upon reasonable request. The data are not publicly available due to privacy restrictions and patient confidentiality considerations.

## Abbreviations

The following abbreviations are used in this manuscript:

AUC	area under the curve
CT	computed tomography
DOPA	dihydroxyphenylalanine
FDG	[^18^F]fluorodeoxyglucose
FH	fumarate hydratase
HIF-1	hypoxia-inducible factor-1
IDH	isocitrate dehydrogenase
IQR	interquartile range
MIBG	metaiodobenzylguanidine
MRI	magnetic resonance imaging
MTV	total metabolic tumor volume
OSEM	ordered subset expectation maximization
PET	positron emission tomography
PHD	prolyl hydroxylase domain-containing protein
PPGLs	pheochromocytomas and paragangliomas
PRRT	peptide receptor radionuclide therapy
ROC	receiver operating characteristic
SD	standard deviation
SDH	succinate dehydrogenase
SRE	skeletal-related event
SSTR	somatostatin receptor
SUV	standardized uptake value
SUVmax	maximum standardized uptake value
TCA	tricarboxylic acid
TLG	total tumor lesion glycolysis
VHL	von Hippel–Lindau

## References

[1] Mete O., Asa S.L., Gill A.J., Kimura N., de Krijger R.R., Tischler A. (2022). Overview of the 2022 WHO classification of paragangliomas and pheochromocytomas. Endocr. Pathol..

[2] Choi Y.M., Sung T.Y., Kim W.G., Lee J.J., Ryu J.S., Kim T.Y., Kim W.B., Hong S.J., Song D.E., Shong Y.K. (2015). Clinical course and prognostic factors in patients with malignant pheochromocytoma and paraganglioma: A single institution experience. J. Surg. Oncol..

[3] Ezzat Abdel-Aziz T., Prete F., Conway G., Gaze M., Bomanji J., Bouloux P., Khoo B., Caplin M., Mushtaq I., Smart J. (2015). Phaeochromocytomas and paragangliomas: A difference in disease behaviour and clinical outcomes. J. Surg. Oncol..

[4] Nölting S., Bechmann N., Taieb D., Beuschlein F., Fassnacht M., Kroiss M., Eisenhofer G., Grossman A., Pacak K. (2022). Personalized management of pheochromocytoma and paraganglioma. Endocr. Rev..

[5] Lenders J.W.M., Kerstens M.N., Amar L., Prejbisz A., Robledo M., Taieb D., Pacak K., Crona J., Zelinka T., Mannelli M. (2020). Genetics, diagnosis, management and future directions of research of phaeochromocytoma and paraganglioma: A position statement and consensus of the Working Group on Endocrine Hypertension of the European Society of Hypertension. J. Hypertens..

[6] Hescot S., Curras-Freixes M., Deutschbein T., van Berkel A., Vezzosi D., Amar L., de la Fouchardière C., Valdes N., Riccardi F., Do Cao C. (2019). Prognosis of malignant pheochromocytoma and paraganglioma (MAPP-Prono Study): A European Network for the Study of Adrenal Tumors retrospective study. J. Clin. Endocrinol. Metab..

[7] Dahia P.L.M., Clifton-Bligh R., Gimenez-Roqueplo A.P., Robledo M., Jimenez C. (2020). Hereditary endocrine tumours: Current state-of-the-art and research opportunities: Metastatic pheochromocytomas and paragangliomas: Proceedings of the MEN2019 workshop. Endocr. Relat. Cancer.

[8] Bechmann N., Moskopp M.L., Ullrich M., Calsina B., Wallace P.W., Richter S., Friedemann M., Langton K., Fliedner S.M.J., Timmers H.J.L.M. (2020). HIF2α supports pro-metastatic behavior in pheochromocytomas/paragangliomas. Endocr. Relat. Cancer.

[9] Eisenhofer G., Deutschbein T., Constantinescu G., Langton K., Pamporaki C., Calsina B., Monteagudo M., Peitzsch M., Fliedner S., Timmers H.J.L.M. (2020). Plasma metanephrines and prospective prediction of tumor location, size and mutation type in patients with pheochromocytoma and paraganglioma. Clin. Chem. Lab. Med..

[10] Eisenhofer G., Huynh T.T., Pacak K., Brouwers F.M., Walther M.M., Linehan W.M., Munson P.J., Mannelli M., Goldstein D.S., Elkahloun A.G. (2004). Distinct gene expression profiles in norepinephrine- and epinephrine-producing hereditary and sporadic pheochromocytomas: Activation of hypoxia-driven angiogenic pathways in von Hippel-Lindau syndrome. Endocr. Relat. Cancer.

[11] Nielsen S.M., Rhodes L., Blanco I., Chung W.K., Eng C., Maher E.R., Richard S., Giles R.H. (2016). Von Hippel-Lindau disease: Genetics and role of genetic counseling in a multiple neoplasia syndrome. J. Clin. Oncol..

[12] Eisenhofer G., Lenders J.W., Linehan W.M., Walther M.M., Goldstein D.S., Keiser H.R. (1999). Plasma normetanephrine and metanephrine for detecting pheochromocytoma in von Hippel-Lindau disease and multiple endocrine neoplasia type 2. N. Engl. J. Med..

[13] Rednam S.P., Erez A., Druker H., Janeway K.A., Kamihara J., Kohlmann W.K., Nathanson K.L., States L.J., Tomlinson G.E., Villani A. (2017). Von Hippel-Lindau and hereditary pheochromocytoma/paraganglioma syndromes: Clinical features, genetics, and surveillance recommendations in childhood. Clin. Cancer Res..

[14] Eisenhofer G., Lenders J.W., Timmers H., Mannelli M., Grebe S.K., Hofbauer L.C., Bornstein S.R., Tiebel O., Adams K., Bratslavsky G. (2011). Measurements of plasma methoxytyramine, normetanephrine, and metanephrine as discriminators of different hereditary forms of pheochromocytoma. Clin. Chem..

[15] Feldman J.M., Blalock J.A., Zern R.T., Shelburne J.D., Gaede J.T., Farrell R.E., Wells S.A. (1979). Deficiency of dopamine-*β*-hydroxylase. A new mechanism for normotensive pheochromocytomas. Am. J. Clin. Pathol..

[16] Takenaka J., Watanabe S., Abe T., Takeuchi S., Hirata K., Kimura R., Ishii H., Wakabayashi N., Majigsuren M., Kudo K. (2025). Urinary dopamine levels can predict the avidity of post-therapy [^131I^]MIBG scintigraphy in unresectable or metastatic pheochromocytomas and paragangliomas: A preliminary clinical study. Pharmaceuticals.

[17] Bodei L., Mueller-Brand J., Baum R.P., Pavel M.E., Hörsch D., O’Dorisio M.S., O’Dorisio T.M., Howe J.R., Cremonesi M., Kwekkeboom D.J. (2013). The joint IAEA, EANM, and SNMMI practical guidance on peptide receptor radionuclide therapy (PRRNT) in neuroendocrine tumours. Eur. J. Nucl. Med. Mol. Imaging.

[18] Shah M.H., Goldner W.S., Benson A.B., Bergsland E., Blaszkowsky L.S., Brock P., Chan J., Das S., Dickson P.V., Fanta P. (2021). Neuroendocrine and adrenal tumors, version 2.2021, NCCN clinical practice guidelines in oncology. J. Natl. Compr. Canc. Netw..

[19] Taïeb D., Hicks R.J., Hindié E., Guillet B.A., Avram A., Ghedini P., Timmers H.J., Scott A.T., Elojeimy S., Rubello D. (2019). European Association of Nuclear Medicine Practice Guideline/Society of Nuclear Medicine and Molecular Imaging Procedure Standard 2019 for radionuclide imaging of phaeochromocytoma and paraganglioma. Eur. J. Nucl. Med. Mol. Imaging.

[20] Han S., Suh C.H., Woo S., Kim Y.J., Lee J.J. (2019). Performance of ^68^Ga-DOTA-conjugated somatostatin receptor–targeting peptide PET in detection of pheochromocytoma and paraganglioma: A systematic review and metaanalysis. J. Nucl. Med..

[21] Fassnacht M., Assie G., Baudin E., Eisenhofer G., de la Fouchardiere C., Haak H.R., de Krijger R., Porpiglia F., Terzolo M., Berruti A. (2020). Adrenocortical carcinomas and malignant phaeochromocytomas: ESMO-EURACAN Clinical Practice Guidelines for diagnosis, treatment and follow-up. Ann. Oncol..

[22] Severi S., Bongiovanni A., Ferrara M., Nicolini S., Di Mauro F., Sansovini M., Lolli I., Tardelli E., Cittanti C., Di Iorio V. (2021). Peptide receptor radionuclide therapy in patients with metastatic progressive pheochromocytoma and paraganglioma: Long-term toxicity, efficacy and prognostic biomarker data of phase II clinical trials. ESMO Open.

[23] Janssen I., Chen C.C., Zhuang Z., Millo C.M., Wolf K.I., Ling A., Lin F.I., Adams K.T., Herscovitch P., Feelders R.A. (2017). Functional imaging signature of patients presenting with polycythemia/paraganglioma syndromes. J. Nucl. Med..

[24] Fischer A., Kloos S., Maccio U., Friemel J., Remde H., Fassnacht M., Pamporaki C., Eisenhofer G., Timmers H.J.L.M., Robledo M. (2023). Metastatic pheochromocytoma and paraganglioma: Somatostatin receptor 2 expression, genetics, and therapeutic responses. J. Clin. Endocrinol. Metab..

[25] Giammarile F., Chiti A., Lassmann M., Brans B., Flux G. (2008). EANM procedure guidelines for ^131^I-meta-iodobenzylguanidine (^131^I-mIBG) therapy. Eur. J. Nucl. Med. Mol. Imaging.

[26] Hiromasa T., Wakabayashi H., Kayano D., Inaki A., Watanabe S., Mori H., Akatani N., Yamase T., Kunita Y., Saito S. (2022). Prognostic factors for refractory pheochromocytoma and paraganglioma after ^131^I-metaiodobenzylguanidine therapy. Ann. Nucl. Med..

[27] Kayano D., Taki J., Fukuoka M., Wakabayashi H., Inaki A., Nakamura A., Kinuya S. (2011). Low-dose ^123^I-metaiodobenzylguanidine diagnostic scan is inferior to ^131^I-metaiodobenzylguanidine posttreatment scan in detection of malignant pheochromocytoma and paraganglioma. Nucl. Med. Commun..

[28] Inaki A., Shiga T., Tsushima Y., Jinguji M., Wakabayashi H., Kayano D., Akatani N., Yamase T., Kunita Y., Watanabe S. (2022). An open-label, single-arm, multi-center, phase II clinical trial of single-dose [^131^I]meta-iodobenzylguanidine therapy for patients with refractory pheochromocytoma and paraganglioma. Ann. Nucl. Med..

[29] Yoshinaga K., Abe T., Okamoto S., Uchiyama Y., Manabe O., Ito Y.M., Tamura N., Ito N., Yoshioka N., Washino K. (2021). Effects of repeated ^131^I-*meta*-iodobenzylguanidine radiotherapy on tumor size and tumor metabolic activity in patients with metastatic neuroendocrine tumors. J. Nucl. Med..

[30] Wakabayashi N., Watanabe S., Abe T., Takenaka J., Hirata K., Kimura R., Sakamoto K., Shinohara N., Kudo K. (2024). Safety and efficacy of multiple-dose versus single-dose MIBG therapy in patients with refractory pheochromocytoma and paraganglioma: A single-center retrospective analysis. Ann. Nucl. Med..

[31] Wakabayashi H., Taki J., Inaki A., Nakamura A., Kayano D., Fukuoka M., Matsuo S., Nakajima K., Kinuya S. (2013). Prognostic values of initial responses to low-dose ^131^I-MIBG therapy in patients with malignant pheochromocytoma and paraganglioma. Ann. Nucl. Med..

[32] Jha A., Taïeb D., Carrasquillo J.A., Pryma D.A., Patel M., Millo C., de Herder W.W., Del Rivero J., Crona J., Shulkin B.L. (2021). High-specific-activity-^131^I-MIBG versus ^177^Lu-DOTATATE targeted radionuclide therapy for metastatic pheochromocytoma and paraganglioma. Clin. Cancer Res..

[33] Timmers H.J., Chen C.C., Carrasquillo J.A., Whatley M., Ling A., Eisenhofer G., King K.S., Rao J.U., Wesley R.A., Adams K.T. (2012). Staging and functional characterization of pheochromocytoma and paraganglioma by ^18^F-fluorodeoxyglucose (^18^F-FDG) positron emission tomography. J. Natl. Cancer Inst..

[34] Takenaka J., Watanabe S., Abe T., Hirata K., Uchiyama Y., Kimura R., Shinohara N., Kudo K. (2023). Prognostic value of [^18^F]FDG-PET prior to [^131^I]MIBG treatment for pheochromocytoma and paraganglioma (PPGL). Ann. Nucl. Med..

[35] Takenaka J., Watanabe S., Abe T., Tsuchikawa T., Takeuchi S., Hirata K., Kimura R., Wakabayashi N., Shinohara N., Kudo K. (2024). Predictive factors of early ^18^F-fluorodeoxyglucose-positron emission tomography response to [^131^I] metaiodobenzylguanidine treatment for unresectable or metastatic pheochromocytomas and paragangliomas. Neuroendocrinology.

[36] Timmers H.J.L.M., Taïeb D., Pacak K., Lenders J.W.M. (2024). Imaging of pheochromocytomas and paragangliomas. Endocr. Rev..

[37] Urso L., Panareo S., Castello A., Ambrosio M.R., Zatelli M.C., Caracciolo M., Tonini E., Valpiani G., Boschi A., Uccelli L. (2022). Glucose metabolism modification induced by radioligand therapy with [177Lu]Lu/[90Y]Y-DOTATOC in advanced neuroendocrine neoplasms: A prospective pilot study within FENET-2016 trial. Pharmaceutics.

[38] Kierans S.J., Taylor C.T. (2021). Regulation of glycolysis by the hypoxia-inducible factor (HIF): Implications for cellular physiology. J. Physiol..

[39] Hirata K., Kobayashi K., Wong K.P., Manabe O., Surmak A., Tamaki N., Huang S.C. (2014). A semi-automated technique determining the liver standardized uptake value reference for tumor delineation in FDG PET-CT. PLoS ONE.

[40] Wahl R.L., Jacene H., Kasamon Y., Lodge M.A. (2009). From RECIST to PERCIST: Evolving considerations for PET response criteria in solid tumors. J. Nucl. Med..

[41] Crona J., Lamarca A., Ghosal S., Welin S., Skogseid B., Pacak K. (2019). Genotype-phenotype correlations in pheochromocytoma and paraganglioma: A systematic review and individual patient meta-analysis. Endocr. Relat. Cancer.

[42] Taïeb D., Sebag F., Barlier A., Tessonnier L., Palazzo F.F., Morange I., Niccoli-Sire P., Fakhry N., De Micco C., Cammilleri S. (2009). ^18^F-FDG avidity of pheochromocytomas and paragangliomas: A new molecular imaging signature. J. Nucl. Med..

[43] Yoshioka K., Nakano Y., Horichi M., Aono D., Takeshita Y., Takamura T. (2025). Metastatic pheochromocytoma/paraganglioma overproducing multiple catecholamines. JCEM Case Rep..

[44] Eisenhauer E.A., Therasse P., Bogaerts J., Schwartz L.H., Sargent D., Ford R., Dancey J., Arbuck S., Gwyther S., Mooney M. (2009). New Response Evaluation Criteria in Solid Tumours: Revised RECIST Guideline (Version 1.1). Eur. J. Cancer.

[45] Nakazawa A., Higuchi T., Oriuchi N., Arisaka Y., Endo K. (2011). Clinical Significance of 2-[18F]Fluoro-2-Deoxy-D-Glucose Positron Emission Tomography for the Assessment of 131I-Metaiodobenzylguanidine Therapy in Malignant Phaeochromocytoma. Eur. J. Nucl. Med. Mol. Imaging.

[46] Laganà M., Habra M.A., Remde H., Almeida M.Q., Cosentini D., Pusceddu S., Grana C.M., Corssmit E.P.M., Bongiovanni A., De Filpo G. (2024). Adverse skeletal related events in patients with bone-metastatic pheochromocytoma/paraganglioma. Eur. J. Cancer.

[47] Ayala-Ramirez M., Palmer J.L., Hofmann M.C., de la Cruz M., Moon B.S., Waguespack S.G., Habra M.A., Jimenez C. (2013). Bone metastases and skeletal-related events in patients with malignant pheochromocytoma and sympathetic paraganglioma. J. Clin. Endocrinol. Metab..

[48] Eisenhofer G., Pacak K., Huynh T.T., Qin N., Bratslavsky G., Linehan W.M., Mannelli M., Friberg P., Grebe S.K., Timmers H.J. (2011). Catecholamine metabolomic and secretory phenotypes in phaeochromocytoma. Endocr. Relat. Cancer.

[49] van der Harst E., de Herder W.W., de Krijger R.R., Bruining H.A., Bonjer H.J., Lamberts S.W., van den Meiracker A.H., Stijnen T.H., Boomsma F. (2002). The value of plasma markers for the clinical behaviour of phaeochromocytomas. Eur. J. Endocrinol..

[50] Satapathy S., Mittal B.R., Bhansali A. (2019). ‘Peptide receptor radionuclide therapy in the management of advanced pheochromocytoma and paraganglioma: A systematic review and meta-analysis’. Clin. Endocrinol..

[51] Pryma D.A., Chin B.B., Noto R.B., Dillon J.S., Perkins S., Solnes L., Kostakoglu L., Serafini A.N., Pampaloni M.H., Jensen J. (2019). Efficacy and safety of high-specific-activity ^131^I-MIBG therapy in patients with advanced pheochromocytoma or paraganglioma. J. Nucl. Med..

[52] Weber M., Schmitz J., Maric I., Pabst K., Umutlu L., Walz M., Herrmann K., Rischpler C., Weber F., Jentzen W. (2022). Diagnostic performance of ^124^I-metaiodobenzylguanidine PET/CT in patients with pheochromocytoma. J. Nucl. Med..

[53] Wang P., Li T., Cui Y., Zhuang H., Li F., Tong A., Jing H. (2023). ^18^ F-MFBG PET/CT is an effective alternative of ^68^ Ga-DOTATATE PET/CT in the evaluation of metastatic pheochromocytoma and paraganglioma. Clin. Nucl. Med..

[54] Gupta G., Pacak K. (2017). Precision medicine: An update on genotype/biochemical phenotype relationships in pheochromocytoma/paraganglioma patients. Endocr. Pract..

[55] Mihai R., De Crea C., Guerin C., Torresan F., Agcaoglu O., Simescu R., Walz M.K. (2024). Surgery for advanced adrenal malignant disease: Recommendations based on European Society of Endocrine Surgeons consensus meeting. Br. J. Surg..

